# Tests on the Inlay Cement Problem

**Published:** 1905-11-15

**Authors:** Joseph Head

**Affiliations:** Philadelphia, Pa.


### TESTS ON THE INLAY CEMENT PROBLEM.

BY JOSEPH HEAD, M.D., D.D.S., PHILADELPHIA, PA.

In porcelain inlay work the cement line arising from the removal of the matrix has always been a subject of general criticism and discussion. Because No. 30 gold foil is one-third the thickness of the 0.001 in. platinum foil, gold is said to make a finished filling that has a cement line one-third the thickness of the cement line of a filling where a platinum matrix is used. The method of inlay making in which a perfect fit is supposed to be obtained by eliminating the thickness of the matrix, implies that all of the cement is squeezed out of the edges, which is obviously absurd if the inlay is to be held in position.

These and other statements have prompted me to undertake experiments for the purpose of solving the following questions:

*A*—(1) What is the minimum thickness of cement capable of being obtained at the edges of an inlay? (2) Is pressure or fineness of cement grit more responsible for obtaining a fine line? (3) Is it of any practical value to maintain pressure for more than a minute while the cement is setting?

*B*—(1) Does a thick or thin line give the greater strength of retention? (2) Is a fine grit or coarse grit cement the stronger?

*C*—(1) What is the adhesive strength of glazed porcelain cemented to smooth ivory? (2) What is the adhesive strength of etched porcelain cemented to smooth ivory? (3) What is the adhesive strength of etched porcelain cemented to undercut ivory? (4) What is the adhesive strength of undercut porcelain cemented to undercut ivory?

To ascertain the minimum thickness of the cement line, pieces of glass one-quarter inch square were cemented to pieces of glasss one-half inch square. These pieces of glass were so true as to permit atmospheric suction when pressed together in a dry state. To facilitate the removal of the cement film, the surface was slightly oiled by rubbing on the skin. Pressures of eight, twenty-five, fifty and one hundred pounds were used with various grits of the Harvard cement as well as with the Ames inlay cement, to see if pressure or fineness of grit was most responsible for the thin cement line. These pressures in half the number of instances were maintained for a minute only and the further setting continued without pressure. With the other half, the pressure was maintained until setting was complete. This was done to see if the maintaining of the pressure for more than one minute was of any practical value in reducing the cement line. The one-quarter inch square film was chosen as a standard cement surface. This is larger than the average surface of a porcelain inlay, but since the film was made under eight, twenty-five, fifty and one hundred pounds pressure, it was considered that one-eighth inch square film was under a pressure of two, six and one-quarter, twelve and one-half and twenty-five pounds respectively. And as from two to six pounds was found from experiment to be the pressure applied to fillings in the mouth, it was considered that definite conclusions could be obtained from eight, twenty-five, fifty and one hundred pounds pressure applied to films one-quarter inch square, and these films would be large enough to measure at several points and give an average thickness. The measurements for thickness were made on the Black amalgam micrometer (*Cosmos*, vol. xxxvii, page 644), and those for pressure on the Black dynomometer (*Cosmos*, vol. xxxvii, page 409). All of these films were allowed to set in a dry state, and it was found that when they were dipped in water the glass pieces separated and the complete film was easily floated off, picked up on a camel's-hair brush and attached to the face plate of the micrometer, when the various readings were obtained by floating the film of cement on a film of water between the measuring terminals. This supporting film of water was found to be 0.0001 inch in thickness in the following way: A reading was taken while the wet film was still adherent to the glass. The cement film was then loosened so that a film of water could creep underneath. The reading was again taken and invariably found to be 0.0001 inch greater. Thus 0.0001 inch will be deducted from every reading given in the table of film measurements unless otherwise stated. The readings of the Black amalgam micrometer were controlled by the Brown & Sharpe micrometer, and while the Brown & Sharpe micrometer could not measure so minutely, in so far as it could measure the figures for both instruments were the same. The pressure generally used in cementing fillings in the mouth was arrived at in the following manner: Porcelain inlays containing about one-eighth inch square of surface were made to fit cavities in a block of ivory. This block of ivory was then placed on scales and the fillings cemented into position. Without looking at the dial, pressures were used such as were felt to be ordinarily used in the mouth, while an assistant noted the readings. It was found that two pounds was the pressure such as would be used where there were frail edges, three to four pounds were safely applied to ordinary fillings, and six pounds seemed the maximum pressure that would ordinarily be applied to a filling of one-eighth inch square surface. This equals one-half pound for frail fillings of one-sixteenth inch square surface. These measurements on the force usually applied in the mouth make the tests of eight and twenty-five pounds pressure the only ones of practical working value, but, as previously stated, films of cement were also made under fifty and one hundred pounds pressure to determine the minimum cement line and to determine whether the pressure under which the cement was set or the fineness of the grit was most responsible for this minimum line.

As previously stated, Ames inlay and Harvard cements were used. Of the Harvard cement four different grits were used. (1) The Harvard coarse, such as is ordinarily used for making fillings and such as was for a long time ordinarily used for cementing inlays. (2) The Harvard inlay cement as prepared for Dr. Jenkins. (3) A finer grit of Harvard which I have had prepared and have used up to the time of these tests. This I shall speak of as the Harvard special. (4) A Harvard cement that I pulverized in an agate mortar until the grit was almost imperceptible to the teeth. This I shall refer to as the Harvard pulverized.

No use was made of a reduced grit of Ames inlay cement, as it was found that any reduction of the powder so hastened the setting as to render the cement unavailable.

Using eight pounds pressure the average thickness of cement line was found to be as follows:

| | |
|---|---|
| Harvard coarse | .0024 in. |
| Harvard inlay | .0017 " |
| Harvard special | .0015 " |
| Harvard pulverized | .0003 " |
| Ames inlay | .0010 " |

Before going over the adhesion tests, let us restate what we wish to determine. (1) Does a thick or thin line give greater strength of retention? (2) Is a fine grit or a coarse grit cement the stronger? (3) What is the adhesive strength of ground porcelain to smooth ivory? (4) What is the adhesive strength of etched porcelain to smooth ivory? (5) What is the adhesive strength of etched porcelain to undercut ivory? (6) What is the adhesive strength of undercut porcelain to undercut ivory.?

The first adhesion tests were made with the glass squares etched with hydrofluoric acid. Pieces one-quarter inch square were cemented to pieces one-half inch square under eight pounds pressure for fifteen minutes, set in water over night. Two specimens of Harvard coarse, three of Harvard pulverized, and two of Ames inlay were thus prepared. In the morning the glasses all fell apart. This result showed that the expansion and contraction of the glass was sufficient to tear it loose from the cement. At first it was thought the tests would have to be made in a culture oven at the normal temperature of the, mouth but tests with a clinical thermometer in the mouth after eating hot soup made it clear that experiments made in an even temperature would not be fair. as it was apparent that a filling might be subjected to hot soup at a temperature of 152 degrees and ice cream at a temperature of 32 degrees, a variation of 120 degrees. Therefore, for the adhesion tests glass was discarded and pieces of porcelain one-quarter inch square and one-sixteenth inch thick, perfectly flat, were cemented to a block of ivory that had been draw-filed flat and smooth. In all of these tests the porcelain and ivory were washed in alcohol and dried before they were cemented together. In the tests where the porcelain was only ground with a wheel the results were so discouraging that they will only be summarized. The porcelain pieces were carefully ground to measure exactly one-quarter inch square to the Brown & Sharpe gage, as it was held that equal cement sufaces should be had if a just comparison was to be obtained.

A number of tests were made under eight pounds pressure and thumb pressure until setting was complete, and some under thumb pressure for a minute, and then released. The Harvard cement was paraffined before being placed in water. The Ames inlay cement was not paraffined, as Dr. Ames says it sets best unprotected. The following morning the pieces of ivory and porcelain were removed from the water, dried with a cloth, and the paraffine and cement carefully cleansed from the edges. The ivory block was then placed in a vise and the pieces of porcelain pulled off sideways with the hook of a spring balance scale. Before each test the dial of the balance was blackened in a gas jet so that the indicator would mark the highest point reached as the cement broke and the pointer flew back. These same spring scales were arranged with nails and board so that they could be used to maintain the required pressure on the cement films as they were setting. The results obtained were so insignificant, there being practically no difference between the maintained pressure, the temporary pressure, the eight pounds pressure and the four pounds or thumb pressure, that for the plain, ground porcelain surfaces I shall only quote those made under thumb pressure released after one minute, and then set in water over night. The tests made with the Harvard coarse gave a braking strain of the following figures: 3, 5, 14, 19, 21, 13, 27, 28, 34, 42 oz.—an average of 20 oz.

The Harvard pulverized gave the following figures: 10, 10, 16, 8, 31, 35, 11, 16, 54, 32 oz.—an average of 22 oz. The Ames cement under similar conditions gave: 0, 0, 64, 20, 10, 6, 0, 0, 10, 32 oz.—an average of 14 oz.

These roughly show a slight superiority of adhesion for the pulverized Harvard, but is not sufficiently conclusive to be of great value. It certainly shows that the adhesion of cement to ground porcelain and to flat, smooth ivory is slight and unreliable. In these tests the cement film seemed to break away equally from the porcelain and ivory.

Having proved that draw-filed ivory and ground porcelain have very little adhesion to cement, these pieces of porcelain were etched for thirty seconds with hydrofluoric acid, washed thoroughly with water, dried, washed in alcohol, and dried again. The blocks of ivory were simply given the ordinary soaking in water to make them as much like the tooth structure as possible, were wiped dry with a cloth, the surfaces washed with alcohol and dried with the air blast. Because the hydrofluoric acid had caused such little adhesion of the cement to the glass, I had doubts as to whether it would cause adhesion to a flat surface of porcelain. I knew that roughening the surface of a rod or deep filling would cause the cement to retain the filling as with undercuts, but there was much doubt in my mind concerning the power of etching to cause adhesion of cement to a flat surface.

Six preliminary tests were made with the one-quarter inch square of etched porcelain cemented to the ivory blocks under eight pounds pressure for one minute and then released—two with Harvard coarse, two with Harvard pulverized, and two with Ames inlay. Next morning, the four Harvard cement films tore away from the ivory at about a 67 oz. pull, remaining firmly adherent to the porcelain, while the Ames cement dropped off the ivory with a pull too small to be measured. This seemed to give a maximum adhesion of the Harvard cement to plain ivory one quarter inch square of a little over four pounds.

It was then seen that if the adhesion of cement to etched porcelain was to be measured, some way would have to be devised to make the cement stick more securely to the ivory. This was done by making undercuts in the ivory a little less than one-quarter inch square. These undercuts were filled with the cement, and the pieces of etched portions pressed upon the overflowing cement kept under eight pounds pressure for one minute, and then released and treated in he usual way. Next morning, when these pieces of porcelain were pulled off, the two etched squares of porcelain broke loose from the Harvard coarse cement at sixteen pounds and twenty-two pounds respectively. Those from the Harvard pulverized at twelve pounds each. The Ames broke at six pounds and three pounds each. In each test there was clean tearing away of the porcelain from the cement. The next tests were identical with those just described, only the old cement in the undercuts was simply filed smooth, and the cleansed porcelain squares cemented under eight pounds pressure for one minute to the old cement and the adjacent ivory that had simply been washed with alcohol and dried. Further tests proved that the fresh cement would stick to the old cement more firmly than it would stick to the etched porcelain.

The following tables and their general averages tell their own story. Tests as previously stated were made under eight pounds pressure for one minute. These same tests were duplicated where the eight pounds pressure was maintained until setting was complete. The Harvard coarse under eight pounds pressure for one minute, released, paraffined, set in water over night and pulled off in the morning, showed that etched porcelain broke away from undercut ivory at

16, 22, 16, 12, 11, 13, 15, 20, 16, 11, 12, 14, 14, 14, 16, 12, 15 and 10 pounds. General average, thirteen pounds, two ounces to one-quarter inch square surface. When the pressure was maintained until setting occurred, the breaking strain was found to give the following figures: 6, 16, 13, 10, 12, 18, 13, 4, 10, 8 and 9 pounds, giving a general average of eleven pounds and ten ounces. This shows that with the Harvard coarse maintaining the pressure did not increase the adhesion. These same tests were made with the Harvard pulverized. Where the eight pounds pressure was maintained for one minute only, we have the following figures: 12, 12, 13, 8, 9, 13, 18, 21 pounds, giving a general average of thirteen pounds, four ounces. Where pressure was maintained until setting was complete, the following were the figures: 16, 12, 25, 24, 28, 17, 14, 13, 24, 16, 17, 13, 17, 12, 19, 16 pounds, giving a general average of seventeen pounds and seven ounces.

With Ames tests where eight pounds pressure at one minute was maintained, the following figures; 6, 3, 13, 13, 0, 17, 11, 3 pounds, giving a general average of eight pounds, four ounces.

Where the pressure was maintained until setting occurred Ames cement gave the following figures: 6, 8, 6, 7, 14, 16, 12, 11, 11, 13, 12, 9, 8, 10, 7, 8, 7, pounds, giving a general average of nine pounds, nine ounces.

### SUMMARY.

**General average for eight Pounds Pressure for one minute only.**

| | |
|---|---|
| Harvard coarse | 13 lb., 2 oz. |
| Harvard pulverized | 13 lb., 4 oz. |
| Ames inlay | 8 lb., 4 oz. |

General average of general averages, eleven pounds, eight ounces.

**General average for eight pounds pressure until setting is complete:**

| | |
|---|---|
| Harvard coarse | 11 lb., 10 oz. |
| Harvard pulverized | 17 lb., 7 oz. |
| Ames Inlay | 9 lb., 9 oz. |

General averages of general averages, twelve pounds, fourteen ounces.

The fact that the Harvard coarse cement does not show greater stress-bearing power when the pressure was maintained until complete setting is possibly explained by the fact that with coarse powder sometimes a particle might be so large as to prevent general pressure. Certainly, with the Harvard cement it is clear that the finer the powder the finer and truer the film and the stronger the adhesion. These tests also show that new cement will stick firmly to old cement; that a thin film does not seem to be as strong as a thick film; that ground porcelain and roughened ivory have little adhesion power, while etched porcelain has great power of adhesion to cement. This seemed at first an anomaly, since etching with hydrofluoric acid has no such power on glass; but a little thought will solve the difficulty. Glass is a homogeneous substance, and etches smoothly and homogeneously, but porcelain, being composed of quartz, feldspar and flux, etches unevenly in pits and undercuts.

The fact that the old cement will adhere to the new cement more firmly than the new cement will adhere to the etched porcelain, makes it feasible to fill undercut cavities with cement until setting occurs, and then to cut out of the cement a simple cavity with clean enamel edges. Thus may difficult filling be converted into a simple filling.

Numerous tests were made with the flat porcelain on which zinc oxide had been fused, to see if such porcelain had greater adhesion to the cement than etched porcelain. When zinc oxide was given just the right fusion one-quarter inch square of porcelain with Harvard pulverized stood as high as thirty pounds pressure, but the next fusing of zinc oxide, to the porcelain, made with equal care, gave sometimes no adhesion at all, or only two or three pounds. Therefore, although at first I had great hope that the principle of partially fusing zinc oxid to the porcelain filling for purposes of adhesion might prove of value, its unreliability finally compelled me to believe that etching with hydrofluoric acid is by all odds the most feasible and trustworthy.

### FILLING TEST.

In order finally to verify the accuracy of these data, it was decided to make a series of ten porcelain fillings of standard size in a piece of ivory, and after they had been cemented into position under various conditions with Ames and pulverized Harvard cements respectively, to push the fillings out of the cavities by a plunger inserted from below, attached to a scale.

The cavities were made and reamed to standard size by a reamer five-sixteenths inch in diameter at the top, two-sixteenths inch deep, and three-sixteenths inch in diameter at the bottom. Holes were carefully bored in the bottom of each cavity through the piece of ivory to make a passage for the plunger. The porcelain fillings were made with platinum molds in the usual way, the holes in the bottom of the cavities being temporarily stopped with wooden plugs. The plunger that fitted easily into these holes was cemented into the inside of a celluloid ring so that the pressure might be made on the plunger by means of a spring balance hook inserted in the ring. When the pressure tests were to be made, the block of ivory containing the cemented inlays was placed upright in a vise. The ring containing the plunger was slipped over it and the plunger inserted in the hole beneath the filling to be tested; then the spring balance hook was placed in the ring, and pressure made until the filling was loosened. The exact pressure reached on the giving way of the fillings was measured by the needle marking a carbon film on the scale as described previously in the other adhesion tests. The first two tests were to obtain the adhesion of perfectly smooth-cut ivory walls to smooth, glazed, closely fitting porcelain. Such fillings in such cavities were cemented into place with pulverized Harvard cement at thumb pressurne for one miute and then allowed to set, care being taken to remove all superfluous cement in the holes under the fillings, so that there would be free movement and apposition to the plunger against the porcelain pieces. The fillings were then paraffined, and placed in water over night. In the morning not one of these fillings stood a pressure of eight ounces, and most of them came out with less. The fillings and cavities were then carefully cleansed of cement with diluted sulphuric acid, and washed in alcohol and dried. The fillings were then re-cemented into position with Ames inlay cement, with a similar result—no filling stood more than eight ounces, and most came out with a pressure of two or three ounces. These tests proved that plain ivory surfaces in apposition with glazed porcelain with a thin film of cement did not make a satisfactory union.

The next two tests were made under exactly similar conditions, only the fillings were etched with hydrofluoric acid for thirty seconds. The walls of the cavities were smooth and without undercuts. The following represent the figures: Harvard pulverized: 37, 28, 47, 96, 20, 30, 34, 66, 64, 88 ounces respectively. General average, three pounds three ounces. Ames inlay: 40, 40, 32, 57, 90, 64, 39, 33, 38, 37 ounces respectively. General average, two pounds, twelve ounces.

In the next tests the conditions were similar to the preceding, only that the cavities instead of being smooth with sloping walls, were deeply undercut. Harvard pulverized: 8½, 9, 11, 15¾, 7, 6, 13, 14 and 18 pounds respectively. General average, eleven pounds, six ounces. Ames inlay: 14, 10, 13½, 7½, 7, 17, 11, 13 and 17½ pounds respectively. General average, twelve pounds, ten ounces.

In the last two tests the same methods were used as in five and six, only the fillings were deeply undercut, so as to resemble small collar buttons, without the slightest attempt being made to fit the cavity except at the margins, and the cavities were more deeply undercut to make as great a cement mass around the fillings as possible. Following are the results: Harvard pulverized, 32, 25, 24, 23, 20, 18, 28, 30 and 27 pounds respectively. General average, twenty-five pounds, seven ounces. Ames inlay: 18, 20, 17½, 10½, 17½ 17½, 33, 26 and 21 pounds respectively. General average, nineteen pounds.

CONCLUSIONS.

These tests and tables indicate: (1, That glazed or ground porcelain with smooth ivory have but little power of adhesion for cement. (2) They show that a thin film of cement is not so strong a bond as a thick mass. (3) The edges of a filling and cavity should be in as close apposition as possible, but wherever feasible, the cement that holds the filling in the cavity should have body. (4) Etching of porcelain with hydrofluoric acid is a valuable means of obtaining adhesion of porcelain with cement on flat, shallow fillings, yet undercuts are to be preferred where they can be obtained, and there is good reason to believe that the best results can be gained by both, undercutting and etching the filling. (5) Fusion of zinc oxid to the porcelain inlay for adhesion purposes is unreliable and not so trustworthy as etching. (6) Fineness of grit in a cement is more responsible for a fine cement line than pressure. (7) Reduction of the grit in Harvard cement adds to the strength and power of adhesion. This is a law that may apply to all cements if they are not thereby made to set too quickly. (8) Maintaining the pressure of setting Harvard pulverized cement after one minute slightly diminishes the cement line and increases the adhesion 24 percent. (9) The cement line of the Harvard cement ordinarily used in inlay work is from one and a half to two and a half times the thickness of platinum inlay foil, 0.001 inch in thickness. (10) Harvard cement grit may be so pulverized that it readily gives a cement line just the thickness of No. 30 gold foil, while its strength and adhesiveness are greatly increased. (11) The flat adhesive tests show such variety or stress that undercuts should be used wherever possible. (12) Where the inlay and cavity are sufficiently deep to allow the final expansion of the cement during crystalization to be exerted on the filling and cavity, maintaining the pressure is only valuable for the sake of apposition, as more pressure is probably obtained by the crystallization of the cement than can be exerted by external pressure

This is shown in the filling tests, where external pressure was maintained for but one minute, and yet much greater retention was had than in any of the flat tests.

For the sake of apposition these tests seem to prove that the maintenance of the pressure for one minute is ample. Still, it is a safe precaution to continue the pressure until setting is complete wherever this is feasible.

Having dealt with the question of fineness of film and adhesiveness and strength of cement, the relative solubility of a coarse or a fine line is still to be determined.

These tests are now in progress but are not sufficiently numerous to be conclusive. Still, it may be of interest to state that in the tests already made, fine lines of Harvard cement were attacked by a 1 percent aqueous solution of lactic acid in two or three days, while they were not attacked by a 1 percent lactic acid solution of saliva during the same time. This apparently shows that the mucus gets into the fine line and acts as a capillary plug, preventing the constant interchange of the solvent fluid.—*Cosmos.*

